# Three-dimensional morphology of the human embryonic brain

**DOI:** 10.1016/j.dib.2015.05.001

**Published:** 2015-05-18

**Authors:** N. Shiraishi, A. Katayama, T. Nakashima, S. Yamada, C. Uwabe, K. Kose, T. Takakuwa

**Affiliations:** a Human Health Science, Graduate School of Medicine, Kyoto University, Kyoto, Japan; b Congenital Anomaly Research Center, Graduate School of Medicine, Kyoto University, Kyoto, Japan; c Institute of Applied Physics, University of Tsukuba, Ibaragi, Japan

## Abstract

The morphogenesis of the cerebral vesicles and ventricles was visualized in 3D movies using images derived from human embryo specimens between Carnegie stage 13 and 23 from the Kyoto Collection. These images were acquired with a magnetic resonance microscope equipped with a 2.35-T superconducting magnet. Three-dimensional images using the same scale demonstrated brain development and growth effectively. The non-uniform thickness of the brain tissue, which may indicate brain differentiation, was visualized with thickness-based surface color mapping. A closer view was obtained of the unique and complicated differentiation of the rhombencephalon, especially with regard to the internal view and thickening of the brain tissue. The present data contribute to a better understanding of brain and cerebral ventricle development.

## Specifications table

**Table:** 

Subject area	Biology
More specific subject area	Developmental anatomy
Type of data	Three-dimensional image
How data was acquired	The morphogenesis of the cerebral vesicles and ventricles was analyzed using images derived from human embryo specimens from the Kyoto Collection, which were acquired with a magnetic resonance microscope equipped with a 2.35-T superconducting magnet.
Data format	Movie (.mov, .mp4)
Experimental factors	Well-preserved human embryos obtained mainly by induced abortion was fixed with formalin and selected for MR microscopic imaging.
Experimental features	Three-dimensional morphogenesis of the human embryonic brain between Carnegie stages 13 and 23 was visualized from MRI.
Data source location	Kyoto and Tsukuba Universities, Japan
Data accessibility	Data is provided in Supplementary materials directly with this article.

## Value of the data

• The three-dimensional human embryonic brain was precisely visualized from MRI data
• The development of the brain was effectively demonstrated using the same scale
• The non-uniform thickness of the brain tissue during development was visualized
• The unique and complicated differentiation of the rhombencephalon was shown

## 1 Data, experimental design, materials and methods

The morphogenesis of the cerebral vesicles and ventricles was analyzed using images derived from 101 human embryo specimens between Carnegie stage 13 and 23 from the Kyoto Collection, which were acquired with a magnetic resonance microscope equipped with a 2.35-T superconducting magnet. The morphology as well as morphometric analysis are provided elsewhere [1].

### 1.1 MR images of human embryo specimens from Kyoto Collection

Approximately 44,000 human embryos comprising the Kyoto Collection are stored at the Congenital Anomaly Research Center of Kyoto University [2,3]. In most of these cases, pregnancy was terminated during the first trimester for socioeconomic reasons under the Maternity Protection Law of Japan. In the laboratory, aborted embryos were measured, examined, and staged using the criteria provided by O’Rahilly and Müller [4]. Approximately 1200 well-preserved human embryos found by two of the authors (C.U. and S. Y.) to be normal on gross examination and between CS13 and CS23 were selected for MR microscopic imaging [3]. The MR images of the embryos were acquired using a super-parallel MR microscope developed with a 2.35 T horizontal bore (40 cm) superconducting magnet [5]. The pulse sequences used for the image acquisition were T_1_-weighted spin echo sequences with 100 ms repetition times and 10–16 ms echo times. The image matrix was 128×128×256 and the size of the voxel varied from 40 μm^3^ to 150 μm^3^. Because the number of signal accumulations was 16 or 24, the total data-acquisition time was 7.3 or 10.9 h. As shown in the previous paper [5], the image intensity of the T_1_-weighted images of the human embryos has a close correlation with that of Nissl staining sections.

The 101 selected embryos were re-examined by two authors (T.N. and T.T.) based on previously described criteria [6]. The samples with apparent deformity and brain shrinkage were excluded from the analysis because prolonged fixation is known to cause MRI artifacts and tissue shrinkage due to dehydration.

### 1.2 Three-dimensional reconstruction and morphometric analysis

Three-dimensional MR image datasets for each embryo were resectioned as sequential 2D images digitally with ImageJ64^™^ (ver. 1.44, National Institutes of Health, Bethesda, Maryland, United States) and saved as Analyze file formats (.hdr, .img).

The brains and ventricles were segmented for 3D reconstruction using FSL View of FMRIB Software Library™ (ver. 4.1.9, Analysis Group, FMRIB, Oxford, UK). Three-dimensional morphology of the brain was computationally reconstructed with Amira™ software (ver. 5.4.0, Visage Imaging, Berlin, Germany).

The regional non-uniform thickness of the brain tissue was visualized using the filter module of the Amira™ software program named surface color mapping by thickness (the thickness of the brain was visualized on the surface with a color scale).
